# Clinical Holistic Medicine: Use and Limitations of the Biomedical Paradigm

**DOI:** 10.1100/tsw.2004.28

**Published:** 2004-05-11

**Authors:** Soren Ventegodt, Mohammed Morad, Eytan Hyam, Joav Merrick

**Affiliations:** ^1^ The Quality of Life Research Center, Teglgårdstræde 4-8, DK-1452 Copenhagen K, Denmark; ^2^ Division of Community Health, Ben Gurion University, Beer-Sheva, Israel; ^3^ Soroka University Medical Center, Clalit Health Services, Faculty of Health Sciences, Ben Gurion University, Beer-Sheva, Israel; ^4^ National Institute of Child Health and Human Development, Office of the Medical Director, Division for Mental Retardation, Ministry of Social Affairs, Jerusalem and Zusman Child Development Center, Division of Pediatrics and Community Health, Ben Gurion University, Beer-Sheva, Israel

**Keywords:** quality of life, QOL, philosophy, human development, holistic medicine, public health, family medicine, Denmark, Israel

## Abstract

The biomedical paradigm is so convincing from a biochemical point of view, and highly efficient in many cases of acute medical problems and emergencies, but unfortunately most patients do not get much better only treated with drugs; they need to do something about their lives themselves. It is highly important for the modern physician to understand the strengths and weaknesses of the modern biomedical paradigm, to understand when and when not to administer drugs to their patients. Often a symptom can be eliminated for a while with drugs, but this is not always good as the patient might need to learn to study the imbalances in life that cause the disturbances and symptoms. For the elderly patient, sometimes life can be extended in spite of the subjective fact that life has come to its end. Sometimes treatment with a drug can teach the patient that quality of life is the responsibility of the physician and not the patient. This learned attitude can give the patient problems later or make them less active in helping themselves (responsibility transfer in the wrong direction).This paper gives a number of examples where medical drugs really are the treatment of choice in general practice and some more doubtful examples of using of the biomedical paradigm.

## INTRODUCTION

In the last 50 years, we have seen the emergence of a new medical science based on molecular biology. It is a highly developed, biochemical science with pharmaceuticals — chemical drugs based on specially designed molecules, often with a specific effect on cellular receptor molecules. Chemically and technologically, this is science at its most advanced stage. On the other hand, many diseases end up being chronic, meaning that they persist in spite of the pharmaceutical treatment. Chronic disease and disability represent a huge burden of ill health and a large, and growing, cost to modern society[1]. The problem of chronic disease has led to the very provocative conclusion that "drugs don't work"[2,3]. It is well known that most drugs need five patients or more treated to demonstrate an effect, meaning that most of the patients are not helped by most of the drugs, a fact that even the medical industry now seems to admit. This leaves us with extremely interesting questions: Who will get well again and who will not? Or put it in another way: What kind of diseases shall we expect to be cured by pharmaceuticals drugs and which diseases would it be wise to treat with other medical strategies, i.e., alternative or holistic therapy on body, mind, and feelings?

## THE STRENGTHS AND WEAKNESSES OF THE BIOMEDICAL PARADIGM

This article provides some success stories of biomedical treatment to demonstrate sufficient examples of successful biomedicine to crystallize the biomedical paradigm in its most useful form. The physician is intervening at the cellular level, often with highly designed and very biologically potent drugs. It is thus possible to regulate aspects of cell functioning, like acid-forming cells can be stopped producing gastric acid (e.g., with Losec), or the communication between cells can be overridden so that the ovaries no longer produce eggs (contraceptive pills), or bacteria can be fought (with drugs like penicillin that inhibit bacterial growth) without any significant impact or damage to the cells of the body in the person treated. Biomedicine is known as "bio", because it applies biochemistry and molecular biology, thereby interfering with the chemistry of life itself.

When you stop and think about it, you will realize that this is really amazing. In principle, biomedicine is highly effective, when it can do this. When you look at it more closely and from a theoretical viewpoint, however, it is less impressive because many of the effects, side effects, and adverse reactions of a pharmaceutical product in a clinical test cannot be explained or predicted on the basis of biomedical theory. The truth is that we do not understand the information systems of the body at the molecular level well enough to do so. Whenever we treat our patients with designed molecules that interfere with the body's intercellular communication, we are not in complete control of the effects and adverse reactions of the drug, in spite of numerous clinical trials in animals and humans . Since we do not have complete knowledge of the underlying causes of a disease — the large biomedical textbooks state with regard to almost any disease that its cause is not understood — and since we do not have complete control of the effects of the drugs prescribed, whether in the short or the long term, we need to keep an eye on how any specific drug will act in a given patient.

Biomedicine has been developed and tested in order to counteract a specific, well-defined pathology. The physician prescribes it for that indication, e.g., depression, allergy, or hypertension. Before it is launched on the market, it has been tested as mentioned above for effects, side effects, and adverse reactions in long series of studies, first in animals and then in humans. It is not possible to know exactly what will happen in the patient until it has been tried. If the medicine works the way it should without any visible damage it will be marketed.

Biomedicine is really good at treating many diseases. But there is a limit to what you can achieve by biochemical means and we should be highly alert to that limit in our medical practice. We should be particularly alert because the problems that many of our patients are facing can often be solved without pills, which cannot solve all their problems after all.

Below are some examples of what we call "perfect biomedicine". These are biomedical cures that we still use in our own medical practice, even when we master a series of holistic treatment techniques, because we acknowledge these cures to be of excellent and unique value to our patients. They consist of antibiotics and other drugs that we apply safely. Sometimes we face problems that we as physicians cannot solve with biomedicine, and must therefore look more closely at the limitations of biomedicine. Both ways of looking gives important information that guide us in our choice of treatment in the clinic.

Let us emphasize, if you should doubt it, that we have absolute faith in science. When a new pharmaceutical has been investigated in randomized studies, which is in principle a thoroughly sound scientific basis, by objective and unbiased researchers and according to standard protocols, we will not hesitate to acknowledge the documented effect. However, often times, many details in the studies performed are open to criticism, especially the danger of scientific bias, when a pharmaceutical company needs to document the efficacy of its own drug. According to recent investigations, such bias appears to be a real threat to the scientific integrity of a study[4] making many patients take pills that actually do not work well on their condition.

When a drug in a clinical study is tested actively for a given symptom, we would generally believe in its effect. But the situation is more complicated; this does not mean, however, that it would be a sensible move in general practice to remove that symptom with this drug, just because it is possible. The symptom suppressed will perhaps just be replaced by a symptom or problem that is deeper, more dangerous, and more difficult to trace. And perhaps the patient has a lesson to learn from the condition, which is why it would be highly problematic to eliminate it, depriving the patient of vital learning.

The modern biomedical physician applies about 100 different medicinal products in his practice. For a pharmacology examination, an excellent test would be to let the student choose the 15 drugs that would suffice in a biomedical practice in the great majority of cases, including antibiotics, analgesics, diuretics, hydrocortisone, anticoagulants, etc. All these drugs have their clinical merits, including from the perspective of holistic medicine. Below, we shall look at some uses of biomedicine with which we fully agree. But first we need to present our holistic perspective so the reader understands why we so willingly admit the drugs to be of limited use: We have an alternative to drugs when it comes to many chronic diseases.

## CLINICAL HOLISTIC MEDICINE IS BASED ON THE LIFE MISSION THEORY AND THE HOLISTIC PROCESS THEORY OF HEALING

The life mission theory[5,6,7,8,9,10] states that everybody has a purpose of life or has a huge talent. Happiness comes from living this purpose and succeeding in expressing the core talent in your life. To do this, it is important to develop as a person into what is known as the natural condition, a condition where the person knows himself and uses all his efforts to achieve what is most important for him.

The holistic process theory of healing[11,12,13,14] and the related quality of life theories[15,16,17] state that the return to the natural state of being is possible whenever the person gets the resources needed for existential healing. The resources needed are, according to the theory, holding in the dimensions: awareness, respect, care, acknowledgment and acceptance with support and processing in the dimensions: feeling, understanding, and letting go of negative attitudes and beliefs. The precondition for holistic healing to take place is trust and the intention for the healing to take place. Existential healing is not a local healing of any tissue, but a healing of the wholeness of the person, making him much more resourceful, loving, and knowledgeable of himself and his own needs and wishes. In letting go of negative attitudes and beliefs, the person returns to a more responsible existential position and an improved quality of life.

The philosophical change of the person healing is often a change towards preferring difficult problems and challenges, instead of avoiding difficulties in life[18,19,20,21,22,23,24,25]. The person who becomes happier and more resourceful often also becomes more healthy, talented, and more able to function[27,28,29]. Interestingly, energy, wisdom, happiness, talent, intelligence, and the human qualities alike are not easily increased with drugs; considering this sad fact, it can hardly be a surprise that health in general, so related to these qualities, is also very difficult to improve with drugs. So let us look at some good examples of when we can use drugs.

## PNEUMONIA AND PENICILLIN

Penicillin is the classical drug and the basis of the biomedical paradigm. We find it a great drug, largely nontoxic, inexpensive, produced from molds that have attacked our food since the beginning of time, and thus presumably have been known to the body for thousands of years. Penicillin is highly active against a range of microorganisms that often affect humans.

Once this is said, penicillin is not really very active in cavities such as the frontal sinus or middle ear, which are almost never reached by the blood distributing the drug in the body. Several studies have shown that penicillin only shortens the course of the disease by a few days on average in the case of inflammation in a cavity. People are happy to be given penicillin. Now they are on medication, things are happening, and all will be well, since everybody knows penicillin and how effective it is. The following cases of pneumonia are trivial from a medical point of view, but show how the daily life of a modern family physician practicing biomedicine is an oft-repeated theme with minor variations.

> **Female, aged 24 years, with pneumonia:** She has suffered from chest constriction for two days. Auscultation of the lungs: ronchi and "dense" sounds. Diagnosis: /Pneumonia/ Prescribe penicillin.

> **Female, aged 86 years, with pneumonia:** Breathing difficulties for 14 days. Feels very ill, is freezing and sweating. Examination: fever, auscultation of the lungs: ronchi, crackles /Pneumonia/ Cannot tolerate penicillin. Prescribe Erythromycin.

The high efficiency of antibiotics gives us another problem, a difficult ethical problem. Perhaps in the case of the old patient, she should be allowed to die from her pneumonia? Before modern antibiotics, pneumonia provided a quick and gentle death — "an old woman's best friend", as Aldous Huxley (1894–1963) said. Today we keep people alive as long as possible often with little consideration for their quality of life, until they develop dementia or a painful cancer, which most people eventually do when they are old enough. Death can be a protracted affair, painful, lonely, and terrible. It can also be quick and merciful. We believe that old people should be afforded a merciful death when their time is up. And there is really no need for the physician to decide when that is. As doctors, we could do the decent thing and ask the old person whether he or she wants help to live on. Many old people are quite clear in their mind and know when their time is up[25].

## OTHER INFECTIONS

> **Female, aged 27 years, with vaginal discharge:** Negative urine stick. Pelvic examination: Cervical motion tenderness of the uterus, otherwise normal. Wet smear: 80% clue cells /Trichomoniasis/ Prescribe Elysol [metronidazole] 500 mg bid for five days. Since she often forgets to take contraceptive pills we talked about switching to an Implanon implant. She will think about it.

Metronidazole preparations are excellent against the bacteria that usually cause this form of lower abdominal infection (*Trichomonas vaginalis*).

> **Female, aged 88 years, with impetigo:** Presents with small pustules and an even rash on the back and with large red erythematous elements on the left arm without sores or blistering. /Impetigo/ Prescribe culture, locally mupirocin (Bactroban) and Azithromycin orally for five days. Should return if the problem persists.

Impetigo is a very common superficial skin infection caused by streptococci, staphylococci, or a combination of both. They can cause impetigo, erysipilas, cellulitis, lymphangitis, furuncles, and abscess. Impetigo can be common in children, but also in adults, where advanced age can lower resistance as in the case above.

> **Male, aged 56 years, with Lyme disease following a tick bite:** Bitten by a tick below the right clavicle. Large erythema 25 × 25 cm, growing every day. /Erythema migrans/ suspected Lyme disease/Prescribe penicillin.

Here we have some probability of saving the man from neuroborreliosis, a borrelian infection in the brain. This is a very unpleasant disease, if left untreated, often ending in brain damage or death[28].

Antibiotics are probably the group of drugs that have gained biomedicine most respect among the general population. It is indeed a great thing to have drugs formulated as eye drops or vagitories to get rid of that terrible itching. All in a few days.

## VITAMIN AND MINERAL SUPPLEMENTS

Another great example of the biomedical paradigm is the highly effective treatment of a vitamin deficiency with oral vitamin pills. Now that we are talking about placebo, it is logical to move on to the vitamin and mineral supplements so often used, probably the clearest example of placebo available today. In our opinion, vitamin and mineral supplements do very little to promote health, objectively speaking. Most scientific studies conclude that minerals and vitamins like vitamin C, widely believed to be beneficial for the general health, make no difference to a person's health[29,30], except in the very few people suffering from a deficiency condition, while other studies show only what seems to be a modest, beneficial effect[31]. So why do people still take these pills?

The health problems affecting the population are rarely due to vitamin or mineral deficiencies, which is why they cannot be cured by these dietary supplements. Nor will the body benefit from getting any more vitamins or minerals than it needs.

Many people take vitamin C when they have a cold. To our knowledge, no scientific study has come up with evidence of the sense in that. Even very large daily supplements of many grams of vitamin C have no guaranteed effect. We can only conclude that lemon tea and grapefruits, rather like rum toddies, are great placebo remedies for the common cold. And if you are now thinking: “but I can feel the effect”, then we will not deny this in any way, but merely ask: “why do you feel that way?” Is it not because a person you trust greatly, perhaps your mother, once told you that it was good?

The very limited insight in biochemistry most people have means that there is an incredible market for this kind of remedy. People believe in the advertising telling them that they will feel fine once they have taken a vitamin pill, and of course they will, because it saves their conscience. Each year, people buy completely useless vitamin pills and mineral supplements for billions of EUROs and dollars.

> **Female, aged 52 years, with iron deficiency:** Blood tests showed iron deficiency and slightly elevated blood sedimentation rate. No other signs of infection. Still chest pain. We try prescribing Modifenac [diclofenac], Multitabs [vitamins and minerals], Ferro Duretter [ferrous salt] bid + physiotherapy. Check iron with new blood test in three months.

The classic: Prescribing iron against iron deficiency. Here the cause of iron deficiency was not discovered. Occult bleeding?

Our general advice on vitamins and minerals is: Unless you suffer from a diagnosed state of deficiency, it is not wise to waste money, time, and energy on vitamin and mineral supplements. If you want to pamper yourself, you can do better and gain more nourishment by putting your money and efforts into cooking.

## IMMUNE SYSTEM DISORDERS

In the following, we present some case studies that describe one of the most frequent causes of visits to the physician: immunological disorders.

> **Boy, aged 4 years, with asthma:** Four-year preventive surveillance examination. Good development, happy, speech well developed, extrovert, sweet and interested. Weight and height catching up, but he is still asthmatic. Recently the parents apparently increased medication to Spirocort [budesonide] 2 × 200 bid.

Many children suffer from asthma, and asthma drugs are very effective. However, we feel that a symptomatic therapy of this kind is not quite satisfactory when instead we might address the root of the problem. To us, this is a question of understanding the child's life and his basic requirements for well being in his family. We see the child as the thermometer of the family and when the child is sick, there is often cause for improvement in the family. Basically, based on the strong covariance of asthma attacks with stress and other psychological factors[32], we see asthma as a psychosomatic disorder with medication as symptomatic therapy. Nevertheless it works really well and with good effect.

> **Girl, aged 8 years, with eczema:** This girl has eczema on her forearm with well-defined, delimited scaly patches, 2 × 2 cm, mostly on left side. Possibly /fungal infection/ Prescribe Brentacort [hydrocortisone, miconazole].

A fungal eczema — we prescribed Brentacort ointment with hydrocortisone and a fungicide.

> **Male, aged 37 years, with neurodermatitis:** He has been suffering from intense itching and a rash around the penis for six months. He scratches it every day with long nails. Wife claims to have same problem. We need to see her, too, as they may be passing it on to each other. The patient has been informed of this. Examination: Intense rash resembling neurodermatitis 6 × 7 cm on the anterior and lateral parts of the penis. Standard check for STDs. No pustules, margins not affected, skin much thickened and eczematous. Scabies suspected, but no visible burrows in the epidermis. Two years ago, elbow region and groin also affected. Most probably a case of /Tinea cruris [jock itch; ringworm]/ /suspected neurodermatitis/. We try prescribing Brentacort [hydrocortisone, miconazole] ointment. If no marked effect within two weeks, the patient should return to the clinic.

Neurodermatitis is very interesting. It means that the patient will scratch where it itches. When he scratches, it itches more, and so he will scratch even more. In the end damage to the skin is complete. It appears thick, uneven, bleeding with deep hollows and old scabs; it can be a terrible sight. It is truly incredible how much damage people with a propensity to scratching can do to themselves. We recall a psychiatric patient who inflicted a hole in his arm right down to the muscle. The cure is simple: Stop scratching! We break the vicious circle with hydrocortisone, which effectively calms the itching.

> **Male, aged 61 years, with pulmonary oedema?:** Breathing difficulties, believes he has water in the lungs. Auscultation of the lungs: normal. Somewhat gasping respiration that is difficult to interpret. No fever. We try increasing Furix [furosemide] 40 mg bid. He should call out-of-hours service or emergency room, if there is no improvement and he deteriorates over the weekend.

Idiopathic pulmonary oedema — which is self-originated without any external cause — is caused by irritation of pulmonary tissues that makes the vessels become permeable and leak, with plasma pouring into the alveoles. Diuretic drugs are highly effective and have saved many patients in that situation.

> **Girl, aged 2 years — Atopic dermatitis — infantile eczema:** Atopical dermatitis. Prescribe hydrocortisone ointment. Mother should treat her for 14 days and wait and see. Should return if the problem persists.

Hydrocortisone ointment is certain to help. It is good and effective, and the fear of adverse reactions in topical use is highly exaggerated. In our opinion it is quite harmless.

> **Male, aged 62 years, with rheumatoid arthritis?:** Complaints of pain in right ankle. Tender, red, swollen and slightly warm corresponding to both ankles, probably mild rheumatoid arthritis. Some points are very tender, especially laterally below the malleolus. We try prescribing Ibuprofen [ibuprofen] with follow-up for further testing.

Ibuprofen and the other NSAIDs are very satisfactory to use. They take it all: fever, inflammation, pain, swellings, redness, heat. And they are tolerated by most patients, they are cheap, and you can take them throughout your life. (But why does the patient contract rheumatoid arthritis?)

> **Female, aged 32 years, with urticaria:** She had urticaria this morning, which has abated substantially by 2 p.m. Prescribe Zyrtec [cetirizine] 10 mg as required. We talked a little about feelings and the correlation between body and mind **—** about feelings controlling the body most of the time.

The antihistaminergic drugs are highly effective against urticaria and similar inflammatory complaints.

> **Female, aged 43 years, with hayfever:** Diprospan [betamethasone] 2 ml IM in the right gluteus maximus muscle. We talk a little about what she can do to minimise her hayfever symptoms.

Our “little talk” will make many biomedical physicians smile because what indeed can you do yourself about hayfever? But the intensity of hayfever may fluctuate considerably. And if the patient can determine when and why she is not bothered by the allergy at all, she might do something to turn these blissful periods into a permanent state. Well, hydrocortisone is truly effective and the season is not that long, so serious adverse reactions rarely occur even at high doses. Personally, we would prefer holistic therapy to Diprospan.

## CONSTIPATION, DIABETES, AND HYPERTENSION

Below is a wide range of problems that can be solved with biomedicine.

> **Female, aged 42 years, with possible gastritis:** In connection with working on shifts, which will come to an end on the 19th this month, the patient vomits at each meal and

> has pain immediately after the meal and before vomiting corresponding to solar plexus. She is also experiencing problems with her boss. No blood in faeces /suspected gastritis / Prescribe Losec [omeprazole]. If the problems continue she should be referred for gastroscopy.

Losec belongs to a group of agents that can inhibit gastric acid secretion almost completely. In many cases, the symptoms disappear as if by magic. Only not in this patient, whom we had to refer to gastroscopy. Losec is a convincing drug; it is rather expensive, but it is genuinely effective and for most patient almost without side effects[28]. It is impressive. (But why do people develop gastritis?)

> **Female, aged 62 years, with hypertension:** BP 150/80, has taken Cozaar [losartan] 50 mg × 1, since half a tablet was too little; the headache returned. Now well controlled.

Hypertension is a dangerous condition, which increases the risk of a stroke. So if not for the adverse reactions of the drug — some rather diffuse and quite common adverse reactions such as fatigue or loss of energy — this therapy would be just fine. Since lowering the blood pressure is important, and since the drugs at our disposal today are not extremely effective, we need to combine two or three at a time. Often there are serious adverse reactions, such as impotence, when beta-blockers also have to be used.

> **Male, aged 43 years, with hypertension:** Complains of large pull in left calf. If there is suspicion of deep vein thrombosis, the right cure will be immobilisation and ultrasound, but as the condition appears to be almost back to normal today with no real deep tenderness, mobilisation is prescribed; the patient walks well on his leg following massaging of calf. Second BP check. Headache. At home: BP measured at 145/95-105; Here, BP = 150/105. He should continue with Norvasc [amlodipine]. Check-up in three months.

This patient will presumably take this medication for half his life. We believe that it is possible to get rid of the elevated blood pressure through personal development, where the patient "grows" out of the problem, so to speak. It is a good alternative for those who are interested in personal development. However, because of his view of life, we cannot reach this patient with holistic therapy. So we will not bother him with it.

> **Male, aged 85 years, with type 2 diabetes:** Diabetes check. Blood glucose 8.9; BP 130/90, well-controlled.

Adult-onset diabetes, now known as type 2 diabetes mellitus (NIDDM), is one of the diseases that we can manage well, and the same applies to type 1 diabetes. That is something to be proud of. Today, people with diabetes have a life, and the complications — the breakdown of nerves, vascular system, eyes — are very limited compared with before biomedicine. This is a great result. (Imagine, though, if young people could completely avoid developing diabetes and having to inject insulin throughout their lives?).

## CONTRACEPTION AND ABORMAL UTERINE BLEEDING

If there is one thing that the biomedical physician is good at, it is preventing unwanted pregnancies. Once the functions of the reproductive organs are understood, it is easy to understand the effect of forms of contraceptives such as condoms, the femidom, intrauterine devices, diaphragms, or contraceptive foams. Abnormal uterine bleeding is often easy to correct with hormones.

## DISCUSSION

Biomedicine is often highly effective when facing acute problems. It is easy to administer and the effort or time for the physician often very limited. Contraceptives are of huge value. Morphine is a sublime help in many cases of terminal disease. Unfortunately, the target of biomedicine is mostly the symptoms and not the real cause of the disease. When the immunological resistance is weakened temporarily and an infection is threatening the life of the patient, an antibiotic can save him by killing the microorganism causing the infection, but even here the cause of the disease is not really the bacteria itself, but the weakened immune response. Most often, the disease is not cured by the biomedical intervention and masking the symptom does not help the patient in the long run. He will get sick again if the immune resistance is not recovered.

For the last 5 decades, physicians have been very optimistic about what could be obtained with a developed biomedicine — from cures to cancer to dramatic prolongation of youth and long vitality. What we generally have seen — with some important exceptions naturally — is more and more specialized drugs, more and more expensive drugs, and more and more potent drugs curing a still minor fragment of the patients treated. The big pharmaceutical companies have been admitting that “the drugs don’t work”[2,3] and huge companies like NovoNordisk are now claiming that prevention of diseases, and not curing them, seems to be the future for the industry[33].

To understand the seriousness of the problem, let us quote the BMJ editor Richard Smith[2]: **“**Now business has outdone parody, and Allen Rogers, worldwide vice president of genetics at Glaxo SmithKline, is reported on the front page of the *Independent* (8 December, p 1) as saying: ‘Our drugs don't work on most patients.’ This is of course not news to physicians. Anybody familiar with the notion of “number needed to treat” (NNT) knows that it is usually necessary to treat many patients in order for one to benefit. NNTs under 5 are unusual, whereas NNTs over 20 are common.”

This is important, so let us give it a second thought, from the emotional perspective of the physician: What will a sincere and ambitious physician feel when he gives a drug to a patient with a NNT of 2 or less? What can he tell his patients, if he is to be honest? With an NNT of 2, he can say “There is a fair chance that you get well again” and he will feel severely frustrated that this is the help he can give his patient, because he wants to make much more than 50% of his patients well. With a number on NNT of 3, he must say “Most likely this drug will not help you, but let give it a try” and he will feel terrible. With a NNT of 5, he must admit “It is highly unlikely that this drug will help you, but there still is a chance, so let us go for that” and he must feel despair. With a NNT of 20, which is common, he must say “I will give you this drug because it helps sometimes, but do not rely on it in any way; this drug cannot even justify a hope” and the ambitious and caring physician will feel hopeless and helpless. Because if you only help 5% of your patients, 95% will leave your clinic without improvement, but instead waste money on the drugs and have severe side effects. But most important, often with a resignation that is as life threatening as the disease itself.

Medicine is evolving, and the hope we had for biomedicine is turning into frustration, skepticism, and for many physicians, despair. This despair can be found in the frustration that physicians cannot really rely on the drugs that were supposed to heal. The drugs will undoubtedly play an important role also for the future medicine, but simple manual medicine like therapeutic touch[34] and the emerging new toolbox of consciousness-based medicine must also be taken into use by the physicians to have a probate set of medical tools for the next century[35,36].

What is important now is that the physician really rely on his own senses. If he experiences that the drugs work, he should use them. As the pharmaceutical companies do almost nothing, for understandable financial reasons, to narrow the indications so that only the patients likely to benefit will get a drug, this must be the task of the physician. And it is not that difficult. It takes some experimentation on the part of the physician, some alternative medical tools to shift among, in order to provide alternatives to the drugs in your practice. This is the purpose in our series of papers on clinical holistic medicine.

So we are not unhappy that biomedicine turned out to be of restricted value to the patients. Actually, an extended toolbox also including consciousness-based medicine will have many advantages, not only for the patient on an individual level, but also for society at large, making its citizens grow into being more conscious, more ethical, more talented, and more socially minded[5,6,7,8,9,10,18,19,20, 21,22,23,24,25].

## CONCLUSION

Drugs do work — but only in special situations and only as a basic rule, where there are background resources to back up the local healing. The drugs have specific activities, which can be used when this specific action is needed. But very often, the symptoms are caused by imbalances, which should be corrected. These imbalances can be attributed to many aspects, such as poor living conditions, so the symptoms have to create or generate a learning process for the patient, so curing the disease that reveals the imbalance in life often prevents the patient from using his opportunity for the learning process. Sometimes a life can be saved with drugs remedying an acute crisis. But many times, this crisis is only a symptom of chronically poor health or even a terminal state of life, and saving the patient which might seem to be a good thing to do today, can be seen as a cruel thing tomorrow. In general, drugs are highly efficient in an acute phase of a disease, but almost inert in the long run. To improve health and cure chronic disease, the whole life and its quality must be improved. This takes more than drugs; this takes a responsible and determined effort on the part of the patient. This is why, in most cases, a holistic approach to the patient is needed if the physician is to bring permanent improvements in quality for life and health to the patient.

The biomedical paradigm is very convincing from a biochemical point of view, and highly efficient in many cases of acute medical problems and emergencies. Unfortunately most patients will not get much better with drugs; they need to do something about their lives themselves.

## ACKNOWLEDGMENTS

This study was supported by grants from IMK Almene Fond. The quality of life research was approved by the Copenhagen Scientific Ethical Committee under number (KF)V.100.2123/91.

## BIOSKETCHES

**Søren Ventegodt, MD,** is the Director of the Quality of Life Research Center in Copenhagen, Denmark. He is also responsible for a Research Clinic for Holistic Medicine in Copenhagen and is a popular speaker throughout Scandinavia. He has published numerous scientific or popular articles and a number of books on holistic medicine, quality of life, and quality of working life. His most important scientific contributions are the comprehensive SEQOL questionnaire, the very short QoL5 questionnaire, the integrated QOL theory, the holistic process theory, the life mission theory, and the Danish Quality of Life Research Survey, 1991–94 in cooperation with the University Hospital of Copenhagen and the late pediatric professor Bengt Zachau-Christiansen. E-mail: ventegodt@livskvalitet.org. Website: http://www.livskvalitet.org

**Mohammed Morad, MD**, is Specialist in Family Medicine, Lecturer in Family Medicine at the National Institute of Child Health and Human Development, Division of Community Health, Ben Gurion University of the Negev and the Medical Director of a large area clinic in the city of Beer-Sheva. He has publications on Bedouin health, health aspects, spiritual health, and aging in persons with intellectual disability, and is a presenter on topics such as health policy and services for the disadvantaged at national and international conferences. E-mail: morad62@barak-online.net

**Eytan Hyam, MD**, is a specialist in family medicine and the Director General of the Soroka University Medical Center, Clalit Health Services, Faculty of Health Sciences, Ben Gurion University, Beer-Sheva, Israel. E-mail: eytanh@clalit.org.il

**Joav Merrick, MD, DMSc,** is Professor of Child Health and Human Development affiliated with the Zusman Child Development Center, Division of Pediatrics and Community Health at the Ben Gurion University, Beer-Sheva, Israel; the Medical Director of the Division for Mental Retardation, Ministry of Social Affairs, Jerusalem; and the Founder and Director of the National Institute of Child Health and Human Development. He has numerous publications in the field of child and human development, rehabilitation, intellectual disability, disability, health, welfare, abuse, advocacy, quality of life, and prevention. Dr. Merrick received the Peter Sabroe Child Award for outstanding work on behalf of Danish Children in 1985 and the International LEGO-Prize ("The Children's Nobel Prize") for an extraordinary contribution towards improvement in child welfare and well being in 1987. E-mail: jmerrick@internet-zahav.net. Website: www.nichd-israel.com
